# Guiding AlphaFold to predict how Munc13‐1 opens Syntaxin‐1

**DOI:** 10.1002/2211-5463.70272

**Published:** 2026-05-28

**Authors:** Madhurima Chattopadhyay, Junjie Xu, Josep Rizo

**Affiliations:** ^1^ Department of Biophysics University of Texas Southwestern Medical Center Dallas TX USA; ^2^ Department of Biochemistry University of Texas Southwestern Medical Center Dallas TX USA; ^3^ Department of Pharmacology University of Texas Southwestern Medical Center Dallas TX USA

**Keywords:** AlphaFold, conformational change, Munc13, neurotransmitter release, SNAREs, Syntaxin

## Abstract

The SNARE proteins syntaxin‐1, synaptobrevin, and SNAP‐25 mediate neurotransmitter release by forming SNARE complexes that fuse synaptic vesicles with the plasma membrane. SNARE complex assembly is orchestrated by Munc18‐1 and Munc13‐1 through a highly regulated pathway that starts with syntaxin‐1 folded into a closed conformation and bound to Munc18‐1. It is well‐established that Munc13‐1 opens syntaxin‐1, likely acting catalytically, and that this step is crucial for neurotransmitter release. However, the underlying molecular mechanism remains unknown because it is difficult to obtain structural information on Munc13‐1‐syntaxin‐1 interactions experimentally. Initial attempts with AlphaFold using the syntaxin‐1 cytoplasmic region yielded structures of Munc13‐1‐syntaxin‐1 complexes but syntaxin‐1 remained closed. Interestingly, when using a shorter syntaxin‐1 fragment designed to destabilize the closed conformation, AlphaFold generated a model of Munc13‐1 bound to an open syntaxin‐1 conformation that explains abundant experimental data and suggests an attractive hypothesis of how Munc13‐1 opens syntaxin‐1. These results indicate that a judicious selection of protein fragments can help AlphaFold to predict structures of kinetic intermediates in complex biomolecular processes.

## Graphical abstract

The syntaxin‐1 Habc‐domain (orange), linker (pink) and SNARE motif (yellow) form a closed conformation that binds to Munc18‐1 (violet) and is opened by the Munc13‐1 MUN domain (cyan) to form the SNARE complex that triggers neurotransmitter release. Judiciously selecting syntaxin‐1 fragments was key to develop an attractive model of how MUN opens syntaxin‐1 to enable synaptobrevin (red) binding using AlphaFold3.

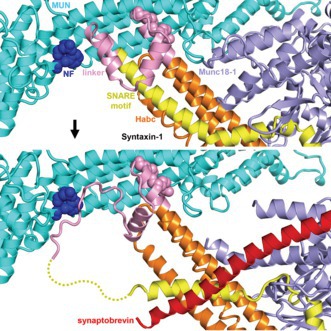

**Abbreviations**
CAPS: calcium‐dependent activator protein for secretion
DAG: diacylglycerol
NMR: nuclear magnetic resonance
NSF: N‐ethylmaleimide sensitive factor
PIP_2_
: phosphatidylinositol 4,5‐bisphosphate
RIM: Rab3 interacting molecule
SNAP: soluble NSF attachment protein
SNAP‐25: synaptosomal associated protein 25 kDa
SNARE: SNAP receptor

The release of neurotransmitters by Ca^2+^‐evoked synaptic vesicle exocytosis mediates neuronal communication and is exquisitely regulated in a wide variety of presynaptic plasticity processes that shape the flow of signals through neural networks, underlying diverse forms of information processing in the brain [1, 2]. Exocytosis requires tethering of the vesicles to the plasma membrane, vesicle priming to a release‐ready state(s), and fast vesicle fusion upon Ca^2+^ influx into a presynaptic terminal [3]. Extensive characterization of the key proteins that govern these steps has led to definition of their functions [4, 5, 6] and allowed biochemical reconstitution of vesicle fusion with these proteins [7, 8, 9]. The SNAP receptors (SNAREs) synaptobrevin, syntaxin‐1, and SNAP‐25 form a tight four‐helix bundle called the SNARE complex via their SNARE motifs [10, 11], bringing the vesicle and plasma membranes together [12] and inducing fusion [13], likely by acting as local detergents [14, 15]. SNARE complexes are disassembled by N‐ethylmaleimide sensitive factor (NSF) and soluble NSF attachment proteins (SNAPs) [10] to recycle the SNAREs [16]. Munc18‐1 and Munc13s organize SNARE complex assembly in an NSF‐SNAP‐resistant manner [7, 17], yielding a spring‐loaded primed state [18] with SNARE complexes bound to the Ca^2+^ sensor synaptotagmin‐1 [19] and to complexins [20, 21]. Release is triggered by Ca^2+^ binding to synaptotagmin‐1 [22], which likely acts as a lever to facilitate structural changes in the SNAREs that are required for fast membrane fusion [23, 24, 25].

The NSF‐SNAP‐resistant pathway that leads to SNARE complex assembly starts with Munc18‐1 bound to a self‐inhibited ‘closed’ conformation of syntaxin‐1 in which its N‐terminal H_abc_ domain [26] binds intramolecularly to the SNARE motif (see domain diagram in Fig. 1) [27, 28]. Munc18‐1 also binds to synaptobrevin [29], forming a template for SNARE assembly [30, 31, 32, 33] while Munc13‐1 bridges the two membranes [8, 34] and facilitates syntaxin‐1 opening to enable formation of the SNARE four‐helix bundle [35, 36, 37, 38]. In addition to playing a crucial role in exocytosis with these activities, Munc13‐1 acts as a master regulator of release through its multidomain architecture. The highly elongated MUN domain of Munc13‐1 [39] mediates syntaxin‐1 opening [35, 36], whereas various other domains bind to distinct agents involved in diverse forms of short‐ and long‐term plasticity such as Ca^2+^, calmodulin, PIP_2_, diacylglycerol (DAG), and Rab3 interacting molecule (RIM) [1, 4, 40, 41, 42, 43, 44] (Fig. 1). Interestingly, these agents likely act at least in part by controlling the syntaxin‐1 opening activity of the MUN domain [45, 46]. The central importance of syntaxin‐1 opening for neurotransmitter release was also emphasized by the fact that syntaxin‐1 bearing a so‐called LE mutation that leaves its conformation constitutively opened (L165A,E166A) [27] enhances the probability of release [47] and partially rescues the phenotypes caused by absence of a wide variety of proteins, including Munc13s, Ca^2+^ channels, neurotransmitter receptors, and other components of the release machinery such as synaptotagmin‐1, RIM, and CAPS [48, 49, 50, 51].

**Fig. 1:**
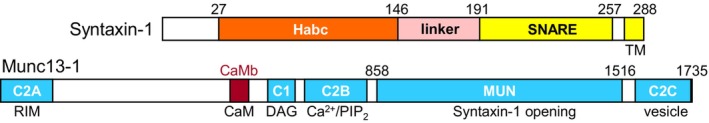
Domain diagrams of syntaxin‐1 and Munc13‐1. SNARE = SNARE motif; CaMb = calmodulin binding domain; TM = transmembrane domain. Selected residue numbers for the rat syntaxin‐1A and Munc13‐1 isoforms are indicated above the diagrams. Binding targets of the Munc13‐1 domains are listed below the corresponding domains.

Despite the wealth of information available on the neurotransmitter release machinery, the structural basis underlying how Munc13‐1 opens syntaxin‐1 has remained enigmatic, likely because binding between Munc13‐1 and syntaxin‐1 is very weak. Thus, Munc13‐1 is believed to act through interactions of its MUN domain with the linker region between the syntaxin‐1 H_abc_ domain and SNARE motif, which are barely detectable by standard NMR experiments at 20 μM concentrations and are weaker than nonspecific binding of the MUN domain to the SNARE motif [35, 36, 37, 38]. Here, we propose a model of how Munc13‐1 opens syntaxin‐1 based on predictions from AlphaFold [52, 53] that required a sensible choice of syntaxin‐1 fragment designed from knowledge of the structures of the Munc18‐1‐closed syntaxin‐1 complex [28] and the syntaxin‐1‐Munc18‐1‐synaptobrevin template complex [33]. This model explains available biochemical and functional data and can now be tested with further experimentation.

## Materials and methods

Predictions with AlphaFold [52] and AlphaFold3 [53] were performed at the AlphaFold server (https://alphafoldserver.com/) using sequences of rat syntaxin‐1A (residues 27–253 or 27–210), rat Munc13‐1 (residues 859–1516), and full‐length rat Munc18‐1. When AlphaFol3 yielded several models that were practically identical (root mean square deviations of about 1 Å for over 10 000 atoms), only a representative structure of the set is presented.

## Results

### Altering AlphaFold predictions by changing protein fragments

AlphaFold provides a powerful tool to predict the structures of proteins and protein complexes [52, 53], particularly when the Predicted Local Distance Difference Test (pLDDT) values are confident or very high (> 70%). However, in our experience, the predictions of protein–protein interfaces are generally less reliable if the affinity is weak. In initial attempts to use AlphaFold to model how Munc13‐1 binds to syntaxin‐1 and thus understand the mechanism of syntaxin‐1 opening, we used fragments corresponding to the Munc13‐1 MUN domain and most of the cytoplasmic region of syntaxin‐1 (Residues 27–253). Since Munc13‐1 is expected to act on the closed syntaxin‐1–Munc18‐1 complex rather than on isolated syntaxin‐1, and Munc18‐1 also binds weakly to the Munc13‐1 MUN domain [35], we built AlphaFold models including Munc18‐1.

**Fig. 2:**
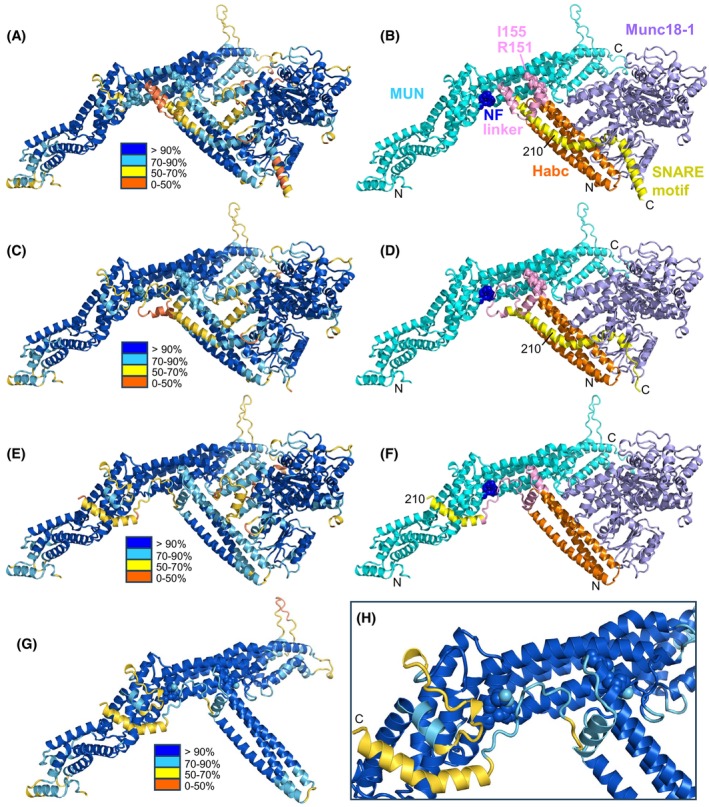
AlphaFold3 models suggesting how Munc13‐1 opens syntaxin‐1. (A‐G) Ribbon diagrams of one of the four nearly identical AlphaFold3 models of the Munc13‐1 MUN domain‐syntaxin‐1(27–253)‐Munc18‐1 complex (model 1) (A, B), the different AlphaFold3 model of the same complex (model 0) (C, D), one of the four nearly identical AlphaFold3 models of the Munc13‐1 MUN‐syntaxin‐1(27–210)‐Munc18‐1 complex (E, F) and one of the five nearly identical AlphaFold3 models of the Munc13‐1 MUN‐syntaxin‐1(27–210) (G). The diagrams are color coded according to pLDDT values as indicated in the insets (A, C, E, G) or the color codes of the domain diagrams of Fig. 1 (B, D, F). N and C indicate N‐ and C‐termini of the MUN domain and the corresponding syntaxin‐1 fragment. (H) Close‐up view of the model of panel (G). In all diagrams, R151, I155 of syntaxin‐1 and the Munc13‐1 NF motif formed by N1128, F1131 are shown as spheres.

All models including Munc18‐1, MUN and syntaxin‐1 (27–253) in initial predictions with AlphaFold [52] and four out of five models built later with AlphaFold3 [53] had analogous architectures, with practically identical binding modes between the MUN domain, syntaxin‐1, and Munc18‐1 complex. We will refer to the four similar models predicted by AlphaFold3 as Models 1–4. A representative member of this set (model 1) is shown in Fig. 2A,B. The structures of Munc18‐1 and syntaxin‐1 within the models and the binding mode between the two proteins were very similar to those observed in the crystal structure of the binary closed syntaxin‐1‐Munc18‐1 complex [28]. In the models, Munc18‐1 interacted with the C‐terminal end of the MUN domain while syntaxin‐1 remained closed and bound to a region close to the center of the MUN domain. Interestingly, residues of syntaxin‐1 that interact with MUN in all these models included R151 and I155, which have been shown to be important for Mun‐syntaxin‐1 binding and for syntaxin‐1 opening by Munc13‐1 [37, 38]. Moreover, syntaxin‐1 binding occurred near the N1128,F1131 dyad (NF motif) of Munc13‐1 that plays a key role in opening syntaxin‐1 [36], although the motif did not contact syntaxin‐1 (Fig. 2B).

The pLDDT values for the structure of the syntaxin‐1 region containing R151,I155 were high, but those for the neighboring region of the syntaxin‐1 linker close to the MUN NF motif were low (Fig. 2A). Note that the linker is unstructured in the SNARE complex [26, 27, 54] and adopts distinct conformations in the closed syntaxin‐1‐Munc18‐1 complex [28] and in the template complex formed by syntaxin‐1, Munc18‐1, and synaptobrevin [33]. The structure of the syntaxin‐1 linker region is thus known to be malleable and is expected to be altered during the events that lead to syntaxin‐1 opening. Therefore, while these AlphaFold models were intriguing because they correlate with some of the biochemical and functional data that are available, they did not shed light on how Munc13‐1 opens syntaxin‐1.

Among the five AlphaFold3 models built with Munc18‐1, MUN, and syntaxin‐1(27–253) to address this question, one of them (referred to as model 0) did reveal a distinct conformation of Residues 171–188 of the syntaxin‐1 linker domain with respect to the structure observed in Models 1–4. Hence, these residues formed a loop that contacts the NF motif of Munc13‐1 in Model 0 (Fig. 2C,D), whereas they adopted a helical hairpin structure in Models 1–4 (e.g., Fig. 2A,B). However, the structure of this loop was not predicted with high confidence and syntaxin‐1 remained closed, with the N‐terminal half of the SNARE motif still in contact with the H_abc_ domain and the C‐terminal half interacting with H_abc_ and Munc18‐1.

We reasoned that AlphaFold3 normally yields energetically stable states. If the action of Munc13‐1 is catalytic, an intermediate of the syntaxin‐1 opening pathway with more extensive syntaxin‐1‐MUN interactions that catalyze opening may not be generated by AlphaFold3 because the intermediate may have higher energy than the initial state. We further reasoned that AlphaFold3 might yield the structure of such an intermediate if the syntaxin‐1 SNARE motif was truncated such that many of the interactions that stabilize the syntaxin‐1 closed conformation and prevent syntaxin‐1 opening were absent. This prediction was also based in part on the assumption that, in the beginning of the syntaxin‐1 opening reaction, structural changes should occur in the linker region and in the N‐terminal region of the syntaxin‐1 SNARE motif. Note that this region interacts with the synaptobrevin SNARE motif N terminus in the template complex while interactions of the C‐terminal half of the syntaxin‐1 SNARE motif with H_abc_ and Munc18‐1 [33] remain similar to those observed in the closed syntaxin‐1‐Munc18‐1 complex [28].

Interestingly, the syntaxin‐1 linker interacted more extensively with the MUN domain in four out of five AlphaFold3 models (Models 0–3) built with the Munc13‐1 MUN domain, Munc18‐1 and a syntaxin‐1 fragment spanning Residues 27–210 (thus containing only the 20 N‐terminal residues of the SNARE motif, i.e., Residues 191–210) (e.g., Fig. 2E,F). Because Munc18‐1 stabilizes the closed conformation, we also used AlphaFold3 to predict the structure of the complex between MUN and syntaxin‐1(27–210) without Munc18‐1 to favor syntaxin‐1 opening. All five models obtained in the absence of Munc18‐1 were practically identical and the structure of the linker region was predicted with high confidence (e.g., Fig. 2G,H), resembling the structure observed in models 0–3 obtained in the presence of Munc18‐1 (Fig. 2E,F). The portion of the SNARE motif remaining in the syntaxin‐1(27–210) fragment bound to the MUN domain in all these models, but the structure of this region was not predicted with high confidence and this interaction is unlikely to occur for the full‐length proteins (see below).

### Proposed mechanism of syntaxin‐1 opening by Munc13‐1

**Fig. 3:**
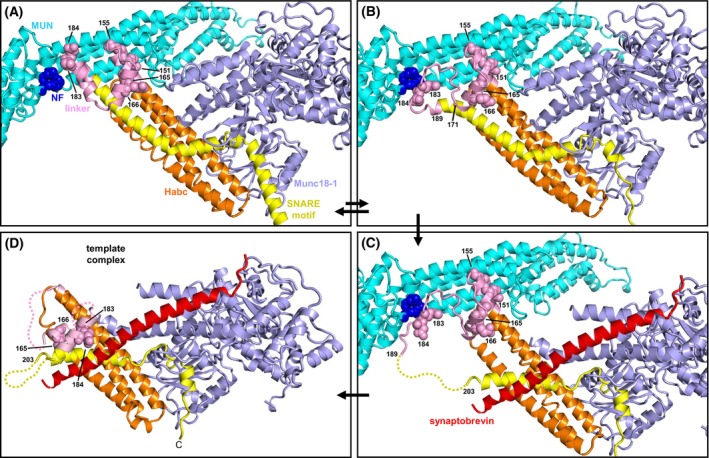
Proposed model of how Munc13‐1 opens syntaxin‐1. (A, B) Close‐up views of the AlphaFold3 models of the complex between the Munc13‐1 MUN domain (cyan), syntaxin‐1 (27–253) (H_abc_ orange, linker pink, SNARE motif yellow) and Munc18‐1 (violet). Panel (A) shows the same model depicted in Fig. 2A,B (model 1), which has syntaxin‐1 in the closed conformation analogous to that observed in its complex with Munc18‐1 [28]. Panel (B) shows the distinct model with residues 171–189 of the syntaxin‐1 linker forming a loop and the syntaxin‐1 conformation slightly opened (model 0, same model as Fig. 2C,D). (C) Model of the Munc13‐1 MUN domain bound to the syntaxin‐1‐Munc18‐1‐synaptobrevin template complex built as a hybrid of one of the cryo‐EM structures of the template complex [33] and the AlphaFold3 model of the Munc13‐1 MUN domain‐syntaxin‐1(27–210)‐Munc18‐1 complex shown in Fig. 2E,F. The MUN domain as well as the syntaxin‐1 linker region and H_abc_ domain are from the AlphaFold3 model, omitting residues 190–210 of syntaxin‐1. Munc18‐1, synaptobrevin (red) and the yellow ribbon corresponding to residues 203–253 of the syntaxin‐1 SNARE motif are from one of the cryo‐EM structures of the syntaxin‐1‐Munc18‐1‐synaptobrevin complex [33]. Residues 190–202 are assumed to be unstructured and are represented by an arbitrary dashed curve. R151, I155, L165, E166, M183 and D184 of syntaxin‐1, as well as the Munc13‐1 NF motif formed by N1128,F1131, are shown as spheres in (A–C). (D) Cryo‐EM structure of the syntaxin‐1‐Munc18‐1‐synaptobrevin template complex used to build the model of (C). Regions of syntaxin‐1 that were not observed in the structure, likely because they were flexible, are also represented by arbitrary dashed curves. (B) and (C) are proposed to be hypothetical intermediates in the pathway that leads from the closed syntaxin‐1‐Munc18‐1 complex to the template complex. See text for additional details.

The models yielded by AlphaFold, together with the available structures of the binary closed syntaxin‐1‐Munc18‐1 complex [28] and the ternary syntaxin‐1‐Munc18‐1‐synaptobrevin template complex [33], suggest an attractive model of the pathway that leads to syntaxin‐1 opening by Munc13‐1 and initiation of SNARE complex assembly (Fig. 3). In this model, the pathway starts when the closed syntaxin‐1‐Munc18‐1 complex binds to the Munc13‐1 MUN domain as observed in most AlphaFold and AlphaFold3 models that we built with syntaxin‐1(27–253), MUN and Munc18‐1 (e.g., Fig. 2A,B; see close‐up in Fig. 3A). Such binding does not involve substantial structural changes in syntaxin‐1, Munc18‐1 or the MUN domain, and is mediated by the region of the syntaxin‐1 linker containing R151,I155. The mode of interaction of this region with the MUN domain is the same in all the AlphaFold models that we generated, and the pLDDT values in this region were high, supporting the validity of this binding mode. Interactions of Munc18‐1 with MUN may cooperate with those involving syntaxin‐1 R151,I155 to form the MUN‐syntaxin‐1‐Munc18‐1 complex.

The structure of the unique AlphaFold3 model obtained with syntaxin‐1(27–253), MUN, and Munc18‐1 shown in Fig. 2C,D (model 0; close‐up in Fig. 3B) suggests that initiation of syntaxin‐1 opening involves a structural rearrangement of the sequence spanning Residues 171–189 of the syntaxin‐1 linker, which initially forms a short helical hairpin (Fig. 3A) and switches to a loop structure (Fig. 3B). This switch is triggered by establishment of more extensive interactions of the 171–189 syntaxin‐1 sequence with the MUN domain, including contacts with the NF motif. In this state, the N terminus of the SNARE motif (adjacent to the linker) starts to separate a little from the H_abc_ domain (compare Fig. 3A and Fig. 3B), thus weakening or losing some interactions that stabilize the closed conformation. It is plausible that this state is less stable than the initial state with fully closed syntaxin‐1 and that there is an equilibrium between the two states, with the slightly open state of Fig. 3B being visited only transiently and normally reverting back to the fully closed state of Fig. 3A.

Nevertheless, during some of the transient visits to the state of Fig. 3B, the MUN domain may establish additional interactions with the syntaxin‐1 linker, up to residue 189, leading to structures resembling the AlphaFold3 models obtained with the syntaxin‐1(27–210) fragment (e.g., Fig. 2E–H). The high pLDDT values for the structure of Residues 171–189 of syntaxin‐1 in the model of Fig. 2G,H support the validity of this interaction. However, the interactions of the SNARE motif N terminus with MUN observed in these models are very unlikely to occur because the other end of the SNARE motif is expected to remain bound to the H_abc_ domain and Munc18‐1, as observed in the synaptobrevin‐Munc18‐1‐syntaxin‐1 template complex [33] (note that the pLDDT values are low for the SNARE motif N terminus in the models of Fig. 2E–H). It seems likely that the additional interactions of the syntaxin‐1 linker with the MUN domain existing in the state of Fig. 2E–H, compared to the state of Fig. 2C,D, may just help to detach the SNARE motif N terminus from the H_abc_ domain. Such detachment may also be favored by the binding of the syntaxin‐1 SNARE motif to the synaptobrevin SNARE motif that leads to the template complex. These ideas are illustrated by the model of Fig. 3C, which was built as a hybrid of the AlphaFold3 model of Fig. 2E,F and one of the cryo‐EM structures of the template complex [33] superimposing their H_abc_ domains. This hybrid model assumes that Residues 190–202 of syntaxin‐1 become unstructured to accommodate the structural changes required for binding of the syntaxin‐1 linker to MUN and for binding between the syntaxin‐1 and synaptobrevin SNARE motifs. Since the syntaxin‐1 linker forms a small four‐helix bundle with the syntaxin‐1 and synaptobrevin SNARE motifs in the template complex, our model proposes that formation of this structure releases syntaxin‐1 from the MUN domain and yields the ternary syntaxin‐1‐Munc18‐1‐synaptobrevin template complex (Fig. 3D).

## Discussion

After the discovery that syntaxin‐1 forms a closed conformation that binds to Munc18‐1 [27, 28] and that this complex forms the starting point for the NSF‐SNAP‐resistant pathway that leads to neurotransmitter release [7, 17], it became clear that syntaxin‐1 opening is a central event for neurotransmitter release. This notion was further emphasized by the finding that the open syntaxin‐1 LE mutant partially rescues defects in release caused by absence of diverse proteins [48, 49, 50, 51]. Moreover, Munc13‐1 was shown to mediate opening of syntaxin‐1 [35, 36], an activity that underlies in part the essential nature of Munc13s for neurotransmitter release [55, 56, 57, 58] and is likely the focal point of regulation of diverse presynaptic plasticity processes [40, 41, 42, 43, 44, 45, 46]. However, elucidating the molecular mechanism underlying syntaxin‐1 opening experimentally has been difficult because of the very weak nature of the syntaxin‐1‐Munc13‐1 interactions that facilitate syntaxin‐1 opening [35, 37, 38]. Note in this context that the Munc13‐1 MUN domain binds with higher affinity to the syntaxin‐1 SNARE motif than to the syntaxin‐1 linker, but strong evidence indicates that the linker‐MUN interactions are key for syntaxin‐1 opening [35, 37, 38] whereas the high promiscuity of the syntaxin‐1 SNARE motif [59, 60] leads to nonspecific binding to MUN. The weak nature of the relevant interactions with syntaxin‐1 suggests that Munc13‐1 acts catalytically, as the limited binding energy associated with weak interactions can lower energy barriers sufficiently to cause dramatic increases in reaction rates [35].

Using a powerful computational tool such as AlphaFold to predict structures of kinetic intermediates of complex biomolecular processes such as SNARE complex assembly is also challenging because the training datasets used by AlpahFold were obtained with stable proteins and complexes. Hence, it is natural that AlphaFold has the tendency to generate structures of stable states. To overcome this tendency and attempt to obtain models of potential intermediates in the syntaxin‐1 opening reaction, we used the truncated syntaxin‐1(27–210) fragment that, compared to syntaxin‐1(27–253), lacks Residues 211–253. This simple idea arose because this portion of the syntaxin‐1 SNARE motif stabilizes the closed conformation and its complex with Munc18‐1, and is unlikely to be involved in interactions with the MUN domain. This reasoning was based not only on the available knowledge of the structure of the closed syntaxin‐1‐Munc18‐1 complex but also on the AlphaFold models that we obtained using the syntaxin‐1(27–253) fragment, which placed Residues 211–253 of syntaxin‐1 far from the MUN domain (e.g., Fig. 2A,B). The fact that this sequence still interacts with the H_abc_ domain and Munc18‐1 in the template complex that results after opening syntaxin‐1 (Fig. 3D) also supported the notion that this sequence does not participate in interactions with the MUN domain.

After many years wondering how Munc13‐1 opens syntaxin‐1, we were amazed when using AlphaFold3 with the syntaxin‐1(27–210) fragment yielded the multiple models represented by Fig. 2E–H because the models suggested a natural mechanism for this process (Fig. 3). The mechanism is particularly attractive because it predicts a pivotal role for R151,I155 in docking syntaxin‐1 on the MUN domain near the NF motif and a vital role for the NF motif in catalyzing syntaxin‐1 opening via interactions with the syntaxin‐1 linker, consistent with the biochemical and functional data showing the importance of syntaxin‐1 R151,I155 and the MUN domain NF motif for syntaxin‐1 opening by Munc13‐1 [37, 38]. The single model with a distorted syntaxin‐1 linker yielded by AlphaFold3 when using syntaxin‐1(27–253), Munc18‐1 and MUN (Model 0, Fig. 2C,D) is also interesting because it suggests an earlier intermediate in the opening reaction. It is noteworthy that this model differs in the syntaxin‐1 linker region from the other four AlphaFold3 models built with syntaxin‐1(27–253), Munc18‐1 and MUN (Models 1–4, represented by Model 1 in Fig. 2A,B), which contained syntaxin‐1 in a closed conformation. These observations lead to our conjecture that the single model of Fig. 2C,D represents an intermediate with lower stability than the state of Fig. 2A,B.

The LE mutation (L165A,E166A) in syntaxin‐1 is generally believed to enhance neurotransmitter release because it destabilizes this conformation and hence facilitates syntaxin‐1 opening [27, 47, 48, 49, 50, 51]. This notion is still consistent with our proposed model, as it predicts that interactions involving L165 and E166 may be remodeled during syntaxin‐1 opening (Fig. 3) but eventually need to be released when the SNARE four‐helix bundle is formed and the syntaxin‐1 linker becomes unstructured [27, 54]. Nevertheless, despite the supporting experimental data, the model of Fig. 3 is still speculative and will need to be further tested. For instance, optical tweezer data suggest that there are some uncertainties regarding the structure of the linker in the template complex [61]. The functional relevance of the conformation of the syntaxin‐1 linker observed in the cryo‐EM structures of the template complex (Fig. 3D) was supported by the disruption of liposome fusion caused by mutations in M183 and D184. However, it is plausible that these effects arise because of the participation of these residues (particularly M183) in interactions with the MUN domain predicted by the AlphaFold3 models (Fig. 3B,C). These observations do not rule out the physiological relevance of the cryo‐EM structures and the pathway proposed in Fig. 3, but raise the possibility that a state resembling that of Fig. 3C might act as the template complex.

As cryo‐EM and computational modeling of protein complexes are highly active areas of research, it is likely that continuous improvements in both areas will help to further characterize the intermediates of the pathway of SNARE complex assembly experimentally and computationally, thus allowing to test and refine the model of Fig. 3. The results described here illustrate the synergy that can emerge by combining human reasoning with artificial intelligence approaches even when these approaches are extremely powerful.

## Conflicts of interest

The authors declare no conflicts interest.

## Author contributions

MC and JR ran the AlphaFold and AlphaFold3 predictions. MC, JX, and JR discussed strategies to run the predictions. JR wrote the paper, and MC and JX revised the paper.

## Data Availability

The models described in this manuscript can be readily generated at the AlphaFold server website (https://alphafoldserver.com/) using the sequences indicated in the methods section. They are also available from the corresponding author upon request.
